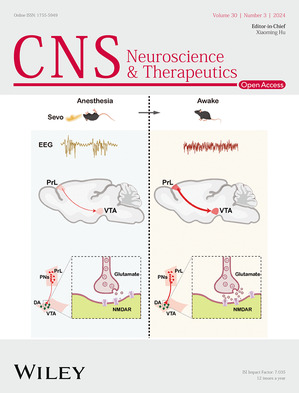# Additional Cover

**DOI:** 10.1111/cns.14731

**Published:** 2024-05-17

## Abstract

The cover image is based on the Original Article *Prelimbic cortical pyramidal neurons to ventral tegmental area projections promotes arousal from sevoflurane anesthesia* by Fuyang Cao et al., https://doi.org/10.1111/cns.14675.